# Alcohol and cannabis use during the COVID-19 pandemic among transgender, gender-diverse, and cisgender adults in Canada

**DOI:** 10.1186/s12889-022-12779-9

**Published:** 2022-03-07

**Authors:** Nibene H. Somé, Mostafa Shokoohi, Kevin D. Shield, Samantha Wells, Hayley A. Hamilton, Tara Elton-Marshall, Alex Abramovich

**Affiliations:** 1 Institute for Mental Health Policy Research, Centre for Addiction and Mental Health, Toronto, ON Canada; 2 Campbell Family Mental Health Research Institute, Centre for Addiction and Mental Health, Toronto, ON Canada; 3 Institute for Clinical Evaluative Sciences, Toronto, ON Canada; 4 Department of Epidemiology and Biostatistics, Schulich School of Medicine and Dentistry, Western University, London, ON Canada; 5 Dalla Lana School of Public Health, University of Toronto, Toronto, ON Canada; 6 World Health Organization/Pan American Health Organization Collaborating Centre in Addiction and Mental Health, Centre for Addiction and Mental Health, Toronto, ON Canada; 7 Department of Psychiatry, University of Toronto, Toronto, ON Canada; 8 School of Psychology, Deakin University, Burwood, Victoria Australia; 9 School of Epidemiology and Public Health, Faculty of Medicine, University of Ottawa, Ottawa, Canada; 10 Department of Health Sciences, Lakehead University, Thunder Bay, Ontario Canada

**Keywords:** COVID-19, Transgender and gender-diverse, Cisgender, Alcohol and cannabis use

## Abstract

**Background:**

This study examined whether heavy episodic drinking (HED), cannabis use, and subjective changes in alcohol and cannabis use during the COVID-19 pandemic differ between transgender and gender-diverse (TGD) and cisgender adults.

**Methods:**

Successive waves of web-based cross-sectional surveys. **Setting**: Canada, May 2020 to March 2021. **Participants**: 6,016 adults (39 TGD, 2,980 cisgender men, 2,984 cisgender women, and 13 preferred not to answer), aged ≥18 years. **Measurements**: Measures included self-reported HED (≥5 drinks on one or more occasions in the previous week for TGD and cisgender men and ≥4 for cisgender women) and any cannabis use in the previous week. Subjective changes in alcohol and cannabis use in the past week compared to before the pandemic were measured on a five-point Likert scale (1: much less to 5: much more). Binary and ordinal logistic regressions quantified differences between TGD and cisgender participants in alcohol and cannabis use, controlling for age, ethnoracial background, marital status, education, geographic location, and living arrangement.

**Results:**

Compared to cisgender participants, TGD participants were more likely to use cannabis (adjusted odds ratio (aOR)=3.78, 95%CI: 1.89, 7.53) and to have reported subjective increases in alcohol (adjusted proportional odds ratios (aPOR)= 2.00, 95%CI: 1.01, 3.95) and cannabis use (aPOR=4.56, 95%CI: 2.13, 9.78) relative to before the pandemic. Compared to cisgender women, TGD participants were more likely to use cannabis (aOR=4.43, 95%CI: 2.21, 8.87) and increase their consumption of alcohol (aPOR=2.05, 95%CI: 1.03, 4.05) and cannabis (aPOR=4.71, 95%CI: 2.18, 10.13). Compared to cisgender men, TGD participants were more likely to use cannabis (aOR=3.20, 95%CI: 1.60, 6.41) and increase their use of cannabis (aPOR=4.40, 95%CI: 2.04, 9.49). There were no significant differences in HED between TGD and cisgender participants and in subjective change in alcohol between TGD and cisgender men; however, the odds ratios were greater than one as expected.

**Conclusions:**

Increased alcohol and cannabis use among TGD populations compared to before the pandemic may lead to increased health disparities. Accordingly, programs targeting the specific needs of TGD individuals should be prioritized.

**Supplementary Information:**

The online version contains supplementary material available at 10.1186/s12889-022-12779-9.

## Background

Disparities in substance use and resulting harms between transgender and gender-diverse (TGD) people (i.e., individuals whose gender identity differs from societal expectations based on their sex assigned at birth) and cisgender people (i.e., individuals whose gender identity aligns with their sex assigned at birth) are well-established [1–5]. Such disparities are due, in part, to TGD individuals having an elevated risk of experiencing mental health problems [5–7], resulting from stigma, discrimination, and violence [8–10].

The COVID-19 pandemic has heightened mental health problems and reduced resilience to chronic stressors among TGD individuals due to delays in providing gender-affirming services, including gender-affirming interventions (e.g., transition-related surgeries, hormone therapy), and to reduced access to TGD support groups [11–13]. Substance use (i.e., alcohol drinking and cannabis use) may represent a coping strategy in response to elevated mental health problems [14–17]. This may result in acute and chronic harms, such as injury, substance use dependence, and death [18–21]. A better understanding of alcohol and cannabis use among TGD people during the pandemic is needed to inform public health interventions since TGD people may be more vulnerable to pandemic harms.

Recent research on the general population has shown an increase in alcohol [22–25] and cannabis [22, 26, 27] use during the pandemic, and that people experiencing high levels of mental health symptoms may be at risk of using substances [28, 29]. This pattern may have increased substance-related hospitalizations and deaths during the pandemic – in Canada, between March and September 2020, hospitalizations and deaths due to substance use increased by 5% and 13% compared to the same period in 2019 [30]. These increases are a pressing concern for the government and public health authorities working on a national recovery plan in response to the COVID-19 pandemic [31, 32]. Additionally, more national data on population health behaviours, such as substance use, are needed to address the needs of the total population [32], including more vulnerable subgroups [33]

Despite the disproportionate impact of the COVID-19 pandemic on the mental health of TGD people (compared to cisgender) [34–36], little research has examined the differential impacts of the pandemic on substance use among TGD versus cisgender individuals. There is a need for more research on this topic to build programs targeting the specific needs of TGD people in response to COVID-19 [37, 38]. Accordingly, this study aimed to assess differences between TGD and cisgender Canadian adults in their respective alcohol and cannabis use during the COVID-19 pandemic. To achieve our objective, we used repeat cross-sectional surveys conducted in Canada examining the impact of COVID-19 on mental health and substance use.

## Methods

### Participants

Data were obtained from seven successive Canada-wide cross-sectional web-based surveys of English-speaking Canadian adults aged ≥18 years. Survey data were collected by Delvinia using a proportional quota sampling methodology to approximate the English speaking population of Canada by age, sex, and region [39]. The surveys were conducted from May 2020 to March 2021: May 8-12, 2020 (Wave 1, *n*=1,005, response rate (RR)=15.9%), May 29-June 1, 2020 (Wave 2, *n*=1,002, RR=17.2%), June 19-23, 2020 (Wave 3, *n*=1,005, RR=16.4%), July 10-14, 2020 (Wave 4, *n*=1,003, RR=13.7%), September 18-22, 2020 (Wave 5, *n*=1,003, RR=17.6%), November 27-December 1, 2020 (Wave 6, *n*=1,003, RR=16.2%), and March 19-23, 2021 (Wave 7, *n*=1000, RR=15.8% (details of RR calculations are in Table S1 of the supplement). A pooled sample of 6,016 participants (Waves 2-7) was analyzed in this study. The questionnaires and data collected are provided in additional files. Wave 1 data were excluded as changes in alcohol and cannabis use were not measured for people who did not report drinking and cannabis use in the past week. The study received approval from the Research Ethics Board at the Centre for Addiction and Mental Health.

### Measures

Gender identity was assessed using the question: “How do you describe your gender identity?” Response options included: “Man,” “Woman,” “Transgender man,” “Transgender woman,” “Two-Spirit,” “Non-binary (genderqueer, gender fluid),” “Questioning/Not sure of my gender identity,” “Identity not listed,” and “Prefer not to answer.” Individuals who self-identified as a man or woman were categorized as cisgender. Individuals who self-identified as transgender, two-spirit, or non-binary and those who selected “Questioning/Not sure of my gender identity” or did not find their gender listed were categorized as TGD. Due to sample size limitations, TGD sub-identities (e.g., transgender man, transgender woman, gender non-binary) were aggregated into one category [40–42]. Participants who responded “prefer not to answer” were excluded from the main analysis. This measure of gender identity may lead to misclassification as TGD participants who self-identified as a man or woman would have been classified as cisgender rather than TGD. We acknowledged that this limitation of the survey could potentially reduce the number of TGD in the sample. An alternative to accurately identify TGD persons in population-based surveys would be, for example, asking two separate questions (i.e., one for current gender identity and another for birth-assigned sex) [43–45].

Heavy episodic drinking (HED) was assessed using the question: “On how many of the past seven days did you drink five/four or more drinks on one occasion?” Cisgender men and TGD participants were asked about ≥5 standard drinks (≥68.0 grams of alcohol) and cisgender women were asked about ≥4 standard drinks (≥54.4 grams of alcohol). We used “5 or more” drinks on one occasion to screen TGD people for heavy drinking as recommended in a recent US study [46]. HED was defined as engaging in at least one HED occasion in the past 7 days. Subjective changes in alcohol use were assessed through the question: “In the past 7 days, did you drink more alcohol, about the same, or less alcohol overall than you did before the COVID-19 pandemic started?” Response options were: 1 (much less), 2 (slightly less), 3 (no change), 4 (slightly more), and 5 (much more).

Cannabis use was derived from the question: “During the past 7 days, on how many days did you use cannabis?” A binary variable was created to reflect any cannabis use (use on one or more days) in the past week versus no cannabis use. Subjective changes in cannabis use were assessed with the question: “In the past 7 days, did you use cannabis more often, about the same, or less often overall than you did before the COVID-19 pandemic started?” As was the case for changes in alcohol use, response options ranged from 1: much less to 5: much more.

The following individual and household covariates were included as confounders in all analyses: age groups (18-39, 40-59, and 60 years or more), ethnoracial background (White, Asian, Black/Indigenous/Arab/Latino, and other ethnicities), marital status (married/living with partner, separated/divorced/widowed, and single), education (high school or less, some post-secondary, college degree, and university degree), geographic location (urban, suburban, and rural), having children under 18 in the household, and whether or not a participant lived with others.

### Statistical analyses

In order to ascertain whether an association existed between gender identity and alcohol/cannabis use during the COVID-19 pandemic, multivariate logistic regression models were used for binary dependent variables (i.e., HED and cannabis use), and ordinal logistic regression models were used for ordinal response variables (i.e., subjective changes in alcohol and cannabis use). Adjusted odds ratios (aORs) and adjusted proportional odds ratios (aPORs) were reported for logistic and ordinal regression models, respectively, adjusting for the above-mentioned covariates. To compare TGD and cisgender individuals in terms of their respective alcohol and cannabis use, alternating reference groups for gender were used in three sets of regression models; that is, TGD participants were compared to: 1) all cisgender participants (both men and women), 2) cisgender men, and 3) cisgender women. Stata (version 16.0) was used for all analyses.

## Results

A total of 39 of 6,003 (0.7%) participants self-identified as TGD, 2,980 (49.6%) as cisgender men, and 2,980 (49.7%) as cisgender women. The TGD group was composed of eight transgender men, four transgender women, eleven two-spirits, nine non-binary (genderqueer and gender fluid), five participants selected “questioning/Not sure of my gender identity”, and two participants did not find their gender listed. Note that in Canada, the estimated percentage of the transgender population, including non-binary individuals, was 0.35% in 2019 [47]. We excluded thirteen participants for not answering the gender identity question. Table 1 presents the distribution of participants’ self-reported consumption of alcohol and cannabis, as well as their socio-demographic characteristics.

**Table 1:** Descriptive statistics: Substance use variables and covariates

	Transgender and gender-diverse individuals		Cisgender individuals (men and women)		Cisgender men		Cisgender women	
	N	%	N	%	N	%	N	%
**N (%)**	**39 (0.7)**		**5964 (99.3)**		**2980 (49.6)**		**2984 (49.7)**	
**Alcohol use variables**								
**Heavy episodic drinking at least once in the past week**								
Yes	33	84.6	4020	67.4	1982	66.5	2038	68.3
No	6	15.4	1,944	32.6	998	33.5	946	31.7
**Subjective changes in alcohol:**								
1: much less alcohol	2	5.3	406	6.9	196	6.7	210	7.1
2: slightly less alcohol	3	7.9	377	6.4	205	7	172	5.8
3: no change	20	52.6	3881	66	1899	64.8	1985	67.3
4: slightly more alcohol	7	18.4	970	16.5	489	16.7	478	16.2
5: much more alcohol	6	15.8	246	4.2	142	4.9	104	3.5
**Cannabis use variables**								
**Cannabis use at least once in the past week**								
Yes	17	43.6	786	13.2	437	14.7	352	11.8
No	22	56.4	5951	86.8	2534	85.3	2628	88.2
**Subjective changes in cannabis:**								
1: much less cannabis	1	2.8	92	1.6	48	1.7	44	1.5
2: slightly less cannabis	1	2.8	119	2.1	72	2.5	47	1.6
3: no change	22	61.1	5091	89.3	2497	87.9	2594	90.7
4: slightly more cannabis	6	16.7	272	4.8	159	5.6	113	4
5: much more cannabis	6	16.7	126	2.2	64	2.3	62	2.2
**Covariates**								
**Age groups years**								
18-39	26	66.7	2308	38.7	1150	38.6	1161	38.9
40-59	10	25.6	1825	30.6	912	30.6	913	30.6
60+	3	7.7	1825	30.6	918	30.8	910	30.5
**Ethnoracial background**								
Asian	9	23.1	1103	18.5	620	20.8	480	16.1
Black/ Indigenous/ Arab/ Latino and other ethnicities	10	25.6	557	9.3	279	9.4	278	9.3
White	19	48.7	4151	69.6	2006	67.3	2143	71.8
**Marital status**								
Married	12	30.8	3769	63.2	2000	67.1	1770	59.3
Separated	7	17.9	722	12.1	257	8.6	466	15.6
Single	19	48.7	1402	23.5	685	23	713	23.9
**Education**								
High school	5	12.8	698	11.7	328	11	367	12.3
Post-secondary	9	23.1	901	15.1	471	15.8	430	14.4
College	7	17.9	1199	20.1	542	18.2	656	22
University	16	41	3119	52.3	1618	54.3	1501	50.3
**Geographic location**								
Urban	16	39	3246	46.6	1728	49.6	1518	43.6
Suburban	15	36.6	2595	37.3	1282	36.8	1313	37.7
Rural	10	24.4	1125	16.1	474	13.6	651	18.7
**Presence of children under 18 years old**								
Yes	8	20.5	1396	23.4	721	24.2	674	22.6
No	31	79.5	4568	76.6	2259	75.8	2310	77.4
**Living arrangement**								
Living with others	27	73	4708	79.2	2379	80.1	2332	78.4
Living alone	10	27	1237	20.8	591	19.9	643	21.6

Table 2 presents the adjusted aORs and aPORs for the associations between gender and HED, cannabis use, and subjective changes in alcohol and cannabis use (Tables S2-S4 in the supplement present information regarding all parameters included in the regression models). No significant differences in HED were observed between the TGD group and cisgender participants (both women and men), cisgender men, and cisgender women; however, the odds ratios were greater than one as expected. TGD participants had higher odds of using cannabis at least once a week (aOR=3.78, 95%CI: 1.89, 7.53) relative to cisgender participants. Similar results were observed when TGD participants were compared to cisgender women (aOR=4.43, 95%CI: 2.21, 8.87) and men (aOR=3.20, 95%CI: 1.60, 6.41). Regarding subjective changes in alcohol and cannabis use compared to before the pandemic, the results showed that TGD participants were at greater odds of reporting subjective increases in their alcohol consumption (i.e., drinking much or slightly more versus no change, slightly less, or much less) relative to cisgender men and women combined (aPOR=2.00, 95%CI: 1.01, 3.95) and cisgender women (aPOR=2.05, 95%CI: 1.03, 4.05). Although the aPOR was greater than one when comparing TGD group to cisgender men regarding subjective changes in alcohol use, it was not statistically significant at 5% level. The TGD group was also at higher odds of reporting an increase in cannabis use than cisgender women and men combined (aPOR=4.56, 95%CI: 2.13, 9.78), cisgender women (aPOR=4.71, 95%CI: 2.18, 10.13), and cisgender men (aPOR=4.40, 95%CI: 2.04, 9.49).

**Table 2:** Associations between TGD status and HED, cannabis use, subjective changes in alcohol and cannabis use

Gender groups	HED at least once in the past week	Cannabis use at least once a week	Subjective change in alcohol use	Subjective change in cannabis use
Transgender and gender-diverse individuals	1.73	3.78^a^	2.00^b^	4.56^a^
	(0.72 - 4.11)	(1.89 - 7.53)	(1.01 - 3.95)	(2.13 - 9.78)
Cisgender individuals (women and men)	Ref	Ref	Ref	Ref
Transgender and gender-diverse individuals	1.68	4.43^a^	2.05^b^	4.71^a^
	(0.70 - 4.01)	(2.21 - 8.87)	(1.03 - 4.05)	(2.18 - 10.13)
Cisgender men	0.94	1.38^a^	1.05	1.07
	(0.84 - 1.05)	(1.19 - 1.62)	(0.95 - 1.17)	(0.90 - 1.27)
Cisgender women	Ref	Ref	Ref	Ref
Transgender and gender-diverse individuals	1.78	3.20^a^	1.94	4.40^a^
	(0.75 - 4.26)	(1.60 - 6.41)	(0.98 - 3.85)	(2.04 - 9.49)
Cisgender women	1.06	0.72^a^	0.95	0.93
	(0.95 - 1.19)	(0.62 - 0.84)	(0.85 - 1.06)	(0.79 - 1.11)
Cisgender men	Ref	Ref	Ref	Ref

^a^ and ^b^ Odds ratios are significant at the 1% and 5% significance level, respectively. 95% confidence intervals in parentheses. Ref: reference category. Subjective changes in alcohol use and changes in cannabis use variables are categorical with five categories: 1 (much less), 2 (slightly less), 3 (no change), 4 (slightly more), and 5 (much more). For these variables, proportional odds ratios were reported. Heavy episodic drinking and the use of cannabis at least once a week are binary variables

Note: Odds ratios and 95% confidence intervals are adjusted for age, marital status, education, ethnoracial background, geographic location, the presence of children, other people in the household, and survey wave indicator variables

Participants who did not respond to the question on gender identity were excluded from the above results (13 participants were excluded). These participants were added into the TGD subsample to conduct a sensitivity analysis. Qualitatively similar results were observed when compared to the main findings (see Table S5 in the supplement).

## Discussion

This study assessed disparities in HED, cannabis use, and subjective alcohol and cannabis use changes during the COVID-19 pandemic between TGD and cisgender participants. Results showed no significant differences in HED between TGD and any of the cisgender groups (i.e., both cisgender men and women, cisgender men or cisgender women), but interestingly the corresponding odds ratios were greater than one as expected. For all comparisons, TGD participants had higher odds of using cannabis. The results also revealed that TGD participants had a higher likelihood of reporting subjective increases in their use of cannabis since the start of the COVID-19 pandemic, compared with cisgender men and women combined, cisgender men, and cisgender women. Additionally, TGD participants were more likely to report subjective increases in their alcohol use during the pandemic than cisgender men and women combined and cisgender women. When compared to cisgender men, the proportional odds ratio of subjective change in alcohol was greater than one but not statistically significant.

These findings should be considered in the context of several limitations. First, the study used a non-probabilistic sample drawn from English-speaking Canadians, and therefore may not be generalizable to the Canadian population. Second, the cross-sectional nature of the study does not allow for causal inference. Third, the relatively small number of TGD respondents prevents the exploration of heterogeneity among TGD subgroups [48–51] and issues of intersectionality. It also resulted in wide confidence intervals for some estimates, thereby making the findings somewhat tentative. The measurement of gender identity in this survey may have impacted the number of TGD respondents. The gender identity question response options may not encompass all gender identities. To accurately classify transgender individuals, experts recommend including a second question measuring sex assigned at birth (i.e., "male" or "female") [44, 45], which was not done in this study. Therefore, some participants may have been grouped incorrectly. Finally, some individuals who do not identify as TGD may have been categorized as such, since those who selected: “Questioning/Not sure of my gender identity” and “Identity not listed” were included in the TGD group. Nevertheless, this preliminary and suggestive study offers useful insights into better understanding disparities in substance use between cisgender and TGD populations during the COVID-19 pandemic.

Our study’s findings are in line with recently published paper using data on Canadian adults [52] and previous research that reported increased risks for harmful drinking and HED [2, 4, 48, 49, 53, 54], and cannabis use [4, 49, 55] among TGD people, relative to their cisgender counterparts. These findings may be partially explained by social stigma and discrimination experienced by TGD individuals, resulting in heightened levels of stress, which may be heightened even further during the pandemic [56]. High levels of stress have been found to be associated with alcohol [57–59] and cannabis use [60–62]. Particularly, among TGD individuals, high levels of physical and psychological gender-related abuse have been associated with higher odds of alcohol and cannabis use [63, 64].

This study found that TGD participants increased their consumption of alcohol and cannabis during the COVID-19 pandemic compared to cisgender participants. These results are consistent with the fact that the implemented lockdown orders have exacerbated mental health symptoms among individuals [65–69], leading to more consumption of alcohol [22–25] and cannabis [22, 26]. The pandemic has exacerbated ongoing mental health disparities for TGD individuals [11, 12, 52, 70] due to reduced access to gender-affirming healthcare and treatment (as non-essential services) and the resulting increased psychological distress [11, 71]. This may have led to an increase in alcohol and cannabis use in TGD populations.

## Conclusions

We identified disparities in HED, cannabis use, and subjective changes in alcohol and cannabis use during the COVID-19 pandemic between TGD and cisgender Canadian adults. Although our study highlights some differences in alcohol and cannabis use during the COVID-19 pandemic between TGD and cisgender people, more research is needed to fully understand disparities in substance use between TGD individuals and cisgender individuals during the COVID-19 pandemic. This will require collecting more data among TGD subgroups (i.e., transgender men, transgender women, two-spirit, and non-binary individuals). Identifying subgroups of TGD individuals at high-risk of engaging in substance use and misuse during the COVID-19 pandemic, and prioritizing gender-affirming medical and mental health care when reopening deferred services, are particularly important to increase general wellbeing among TGD individuals during and after the pandemic.

## Supplementary Information

**Additional file 1:** **Table S1.** Survey interviews information and response rate calculations. **Table S2.** Full estimation: Logistic and ordinal logistic regressions of heavy episodic drinking, cannabis use at least once a week, and change in alcohol and cannabis use on transgender and gender-diverse status with cisgender participants (both men and women) as reference group. **Table S3.** Full estimation: Logistic and ordinal logistic regressions of heavy episodic drinking, cannabis use at least once a week, and subjective change in alcohol and cannabis use on transgender and gender-diverse status with cisgender women as reference group. **Table S4.** Full estimation: Logistic and ordinal logistic regressions of heavy episodic drinking, cannabis use at least once a week, and subjective change in alcohol and cannabis use on transgender and gender-diverse status with cisgender men as reference group. **Table S5.** Sensitivity analysis: Logistic and ordinal logistic regressions of heavy episodic drinking, cannabis use at least once a week, and subjective change in alcohol and cannabis use on transgender or gender-diverse status with participants who did not answer the gender identity question. **Table S6.** Full estimation: Logistic and ordinal logistic regressions of heavy episodic drinking, cannabis use at least once a week, and subjective change in alcohol and cannabis use on transgender and gender-diverse status with cisgender participants (both men and women) as reference group. The six categories of age groups were used in this model. **Table S7.** Full estimation: Logistic and ordinal logistic regressions of heavy episodic drinking, cannabis use at least once a week, and subjective change in alcohol and cannabis use on transgender and gender-diverse status with cisgender women as reference group. The six categories of age groups were used in this model. **Table S8.** Full estimation: Logistic and ordinal logistic regressions of heavy episodic drinking, cannabis use at least once a week, and subjective change in alcohol and cannabis use on transgender and gender-diverse status with cisgender men as reference group. The six categories of age groups were used in this model.

**Additional file 2.** Survey Questionnaire.

**Additional file 3.** Survey data.

## Data Availability

All data generated or analyzed during this study are included in this published article [and its supplementary information files]. Data are also publicly available for download at: http://www.delvinia.com/coronavirus/.

## Abbreviations

HED: Heavy episodic drinking
COVID-19: Coronavirus disease 2019
TGD: Transgender and gender-diverse
aOR: Adjusted odds ratio
aPOR: Adjusted proportional odds ratios
CI: Confidence interval
RR: Response rate
