# Supplementary material for: Alcohol and cannabis use during the COVID-19 pandemic among transgender, gender-diverse, and cisgender adults in Canada
Source: BMC Public Health. 2022 Mar 7;22:452. doi: 10.1186/s12889-022-12779-9 (PMC8899793; doi:10.1186/s12889-022-12779-9)
Supplement: Supplementary file 1 — Additional file 1: Table S1. Survey interviews information and response rate calculations. Table S2. Full estimation: Logistic and ordinal logistic regressions of heavy episodic drinking, cannabis use at least once a week, and change in alcohol and cannabis use on transgender and gender-diverse status with cisgender participants (both men and women) as reference group. Table S3. Full estimation: Logistic and ordinal logistic regressions of heavy episodic drinking, cannabis use at least once a week, and subjective change in alcohol and cannabis use on transgender and gender-diverse status with cisgender women as reference group. Table S4. Full estimation: Logistic and ordinal logistic regressions of heavy episodic drinking, cannabis use at least once a week, and subjective change in alcohol and cannabis use on transgender and gender-diverse status with cisgender men as reference group. Table S5. Sensitivity analysis: Logistic and ordinal logistic regressions of heavy episodic drinking, cannabis use at least once a week, and subjective change in alcohol and cannabis use on transgender or gender-diverse status with participants who did not answer the gender identity question. Table S6. Full estimation: Logistic and ordinal logistic regressions of heavy episodic drinking, cannabis use at least once a week, and subjective change in alcohol and cannabis use on transgender and gender-diverse status with cisgender participants (both men and women) as reference group. The six categories of age groups were used in this model. Table S7. Full estimation: Logistic and ordinal logistic regressions of heavy episodic drinking, cannabis use at least once a week, and subjective change in alcohol and cannabis use on transgender and gender-diverse status with cisgender women as reference group. The six categories of age groups were used in this model. Table S8. Full estimation: Logistic and ordinal logistic regressions of heavy episodic drinking, cannabis use at least once a week, and [file 12889_2022_12779_MOESM1_ESM.docx]

**Supplement**

Table S1: Survey interviews information and response rate calculations

|  | **WAVE 1** *[May 8-12]* | **WAVE 2** *[May 29-Jun 1]* | **WAVE 3** *[June 19-23]* | **WAVE 4** *[July 10-14]* | **WAVE 5** *[Sept 18-22]* | **WAVE 6** *[Nov 27-Dec 1]* | **WAVE 7** *[Marc 19-23]* | ***Pooled Data*: WAVES 2-7** |
| --- | --- | --- | --- | --- | --- | --- | --- | --- |
| ***INTERVIEWS*** | *Total* | *Total* | *Total* | *Total* | *Total* | *Total* | *Total* | *Total* |
| Complete | 1005 | 1002 | 1005 | 1003 | 1003 | 1003 | 1000 | 6016 |
| Incomplete | 109 | 129 | 114 | 112 | 138 | 124 | 130 | 747 |
| Screened | 65 | 89 | 76 | 100 | 112 | 87 | 107 | 571 |
| QuotaFull | 203 | 213 | 131 | 150 | 145 | 135 | 119 | 893 |
| Error | 0 | 0 | 0 | 0 | 0 | 0 | 0 | 0 |
| Total interviews started (*sum of previous cells*) | 1382 | 1433 | 1326 | 1365 | 1398 | 1349 | 1356 | 8227 |
| Invites sent | 6577 | 6130 | 6336 | 7575 | 5962 | 6407 | 6552 | 38962 |
| ***RESPONSE RATE*** | ***%*** | ***%*** | ***%*** | ***%*** | ***%*** | ***%*** | ***%*** | ***%*** |
| RR based on #completed interviews/estimated # eligible respondents [i.e., completed / (sent-quotafull-screened)] | 15.93 | 17.19 | 16.40 | 13.69 | 17.58 | 16.22 | 15.81 | 16.04 |

Table S2: Full estimation: Logistic and ordinal logistic regressions of heavy episodic drinking, cannabis use at least once a week, and change in alcohol and cannabis use on transgender and gender-diverse status with cisgender participants (both men and women) as reference group.

|  | HED at least once in the past week | Cannabis use at least once a week | Subjective change in alcohol use | Subjective change in cannabis use | |
| --- | --- | --- | --- | --- | --- |
|  |  |  |  |  | |
| Transgender and gender-diverse | 1.73 | 3.78*** | 2.00** | 4.56*** | |
|  | (0.72 - 4.11) | (1.89 - 7.53) | (1.01 - 3.95) | (2.13 - 9.78) | |
| Cisgender (men and women) | Ref | Ref | Ref | Ref | |
|  |  |  |  |  | |
| Living with others | 0.99 | 1.19 | 1.03 | 0.87 | |
|  | (0.82 - 1.20) | (0.93 - 1.52) | (0.87 - 1.23) | (0.66 - 1.15) | |
| Presence of children | 1.09 | 0.96 | 1.27*** | 1.03 | |
|  | (0.94 - 1.27) | (0.80 - 1.17) | (1.10 - 1.46) | (0.83 - 1.28) | |
| High school | 0.48*** | 0.90 | 1.20** | 0.98 | |
|  | (0.39 - 0.58) | (0.73 - 1.12) | (1.00 - 1.42) | (0.75 - 1.29) | |
| Post-secondary | 1.04 | 1.18 | 1.01 | 0.98 | |
|  | (0.92 - 1.18) | (0.99 - 1.40) | (0.89 - 1.13) | (0.82 - 1.18) | |
| University | 1.25*** | 1.02 | 0.94 | 0.79* | |
|  | (1.06 - 1.48) | (0.81 - 1.30) | (0.80 - 1.10) | (0.61 - 1.02) | |
| Asian | 0.94 | 0.52*** | 1.14* | 0.64*** | |
|  | (0.82 - 1.09) | (0.43 - 0.62) | (1.00 - 1.31) | (0.52 - 0.79) | |
| White | 0.64*** | 0.24*** | 0.82** | 0.41*** | |
|  | (0.54 - 0.74) | (0.19 - 0.31) | (0.70 - 0.95) | (0.32 - 0.52) | |
| Urban | 0.99 | 1.36** | 1.18 | 1.40** | |
|  | (0.80 - 1.23) | (1.01 - 1.84) | (0.96 - 1.44) | (1.01 - 1.93) | |
| Rural | 0.65*** | 1.02 | 1.25*** | 1.45*** | |
|  | (0.54 - 0.78) | (0.82 - 1.27) | (1.06 - 1.47) | (1.12 - 1.87) | |
| Age 40-59 | 1.04 | 0.97 | 0.92 | 0.92 | |
|  | (0.86 - 1.26) | (0.74 - 1.27) | (0.77 - 1.10) | (0.69 - 1.23) | |
| Age 60+ | 1.01 | 1.02 | 0.90 | 0.93 | |
|  | (0.84 - 1.22) | (0.78 - 1.33) | (0.75 - 1.07) | (0.69 - 1.24) | |
| Separated | 1.27** | 0.94 | 0.72*** | 0.84 | |
|  | (1.05 - 1.54) | (0.71 - 1.23) | (0.60 - 0.87) | (0.62 - 1.12) | |
| Married | 1.20* | 1.34** | 0.80** | 1.32* | |
|  | (0.99 - 1.45) | (1.03 - 1.73) | (0.66 - 0.96) | (1.00 - 1.75) | |
| Wave 2 | 1.32*** | 1.10 | 0.67*** | 0.90 | |
|  | (1.09 - 1.61) | (0.85 - 1.43) | (0.56 - 0.81) | (0.67 - 1.20) | |
| Wave 3 | 0.99 | 1.19 | 1.03 | 0.87 | |
|  | (0.82 - 1.20) | (0.93 - 1.52) | (0.87 - 1.23) | (0.66 - 1.15) | |
| Wave 4 | 1.09 | 0.96 | 1.27*** | 1.03 | |
|  | (0.94 - 1.27) | (0.80 - 1.17) | (1.10 - 1.46) | (0.83 - 1.28) | |
| Wave 5 | 1.48*** | 1.05 | 0.72*** | 1.09 | |
|  | (1.19 - 1.83) | (0.81 - 1.36) | (0.59 - 0.87) | (0.80 - 1.48) | |
| Wave 6 | 1.21* | 1.00 | 0.85* | 1.30* | |
|  | (1.00 - 1.46) | (0.78 - 1.27) | (0.71 - 1.02) | (0.98 - 1.71) | |
| Cutpoint1 |  |  | 0.08*** | 0.01*** | |
|  |  |  | (0.06 - 0.10) | (0.01 - 0.02) | |
| Cutpoint2 |  |  | 0.16*** | 0.02*** | |
|  |  |  | (0.12 - 0.21) | (0.02 - 0.04) | |
| Cutpoint3 |  |  | 4.18*** | 9.56*** | |
|  |  |  | (3.17 - 5.50) | (6.21 - 14.71) | |
| Cutpoint4 |  |  | 25.06*** | 31.97*** | |
|  |  |  | (18.63 - 33.70) | (20.32 - 50.31) | |
| Constant | 5.02*** | 0.28*** |  |  | |
|  | (3.72 - 6.77) | (0.20 - 0.41) |  |  | |
|  |  |  |  |  | |
| Observations | 5,982 | 5,970 | 5,900 | 5,719 | |
| Pseudo R-squared | 0.0495 | 0.0537 | 0.00971 | 0.0188 | |
| **Legend**: *** and ** Odds ratios are significant at the 1% and 5% significance level, respectively. 95% confidence intervals in parentheses. Ref: reference category. | | | | |  |

Table S3: Full estimation: Logistic and ordinal logistic regressions of heavy episodic drinking, cannabis use at least once a week, and subjective change in alcohol and cannabis use on transgender and gender-diverse status with cisgender women as reference group.

|  | HED at once in the past week | Cannabis use at least once a week | Subjective change in alcohol use | Subjective change in cannabis use |
| --- | --- | --- | --- | --- |
| Transgender and gender-diverse | 1.68 | 4.43*** | 2.05** | 4.71*** |
|  | (0.70 - 4.01) | (2.21 - 8.87) | (1.03 - 4.05) | (2.18 - 10.13) |
| Cisgender men | 0.94 | 1.38*** | 1.05 | 1.07 |
|  | (0.84 - 1.05) | (1.19 - 1.62) | (0.95 - 1.17) | (0.90 - 1.27) |
| Cisgender women | Ref | Ref | Ref | Ref |
|  |  |  |  |  |
| Living with others | 0.99 | 1.22 | 1.04 | 0.87 |
|  | (0.82 - 1.19) | (0.95 - 1.56) | (0.87 - 1.23) | (0.66 - 1.15) |
| Presence of children | 1.09 | 0.96 | 1.27*** | 1.03 |
|  | (0.94 - 1.27) | (0.79 - 1.16) | (1.10 - 1.46) | (0.82 - 1.28) |
| High school | 0.48*** | 0.91 | 1.20** | 0.98 |
|  | (0.39 - 0.58) | (0.73 - 1.13) | (1.00 - 1.42) | (0.75 - 1.29) |
| Post-secondary | 1.05 | 1.16* | 1.00 | 0.98 |
|  | (0.92 - 1.18) | (0.98 - 1.38) | (0.89 - 1.13) | (0.81 - 1.18) |
| University | 1.25** | 1.05 | 0.94 | 0.79* |
|  | (1.05 - 1.47) | (0.83 - 1.33) | (0.81 - 1.10) | (0.61 - 1.03) |
| Asian | 0.95 | 0.51*** | 1.14* | 0.64*** |
|  | (0.82 - 1.09) | (0.42 - 0.61) | (1.00 - 1.30) | (0.52 - 0.79) |
| White | 0.64*** | 0.23*** | 0.81*** | 0.41*** |
|  | (0.54 - 0.75) | (0.18 - 0.30) | (0.70 - 0.95) | (0.32 - 0.52) |
| Urban | 0.98 | 1.42** | 1.19 | 1.41** |
|  | (0.79 - 1.22) | (1.05 - 1.91) | (0.97 - 1.45) | (1.02 - 1.95) |
| Rural | 0.65*** | 1.00 | 1.25*** | 1.44*** |
|  | (0.54 - 0.78) | (0.80 - 1.24) | (1.06 - 1.47) | (1.12 - 1.87) |
| Age 40-59 | 1.04 | 0.97 | 0.92 | 0.92 |
|  | (0.86 - 1.26) | (0.74 - 1.28) | (0.77 - 1.10) | (0.69 - 1.23) |
| Age 60+ | 1.01 | 1.02 | 0.90 | 0.93 |
|  | (0.84 - 1.22) | (0.78 - 1.33) | (0.75 - 1.07) | (0.69 - 1.24) |
| Separated | 1.27** | 0.94 | 0.72*** | 0.84 |
|  | (1.05 - 1.54) | (0.71 - 1.23) | (0.60 - 0.87) | (0.62 - 1.12) |
| Married | 1.19* | 1.34** | 0.80** | 1.32* |
|  | (0.99 - 1.45) | (1.04 - 1.74) | (0.66 - 0.96) | (1.00 - 1.76) |
| Wave 2 | 1.32*** | 1.10 | 0.67*** | 0.90 |
|  | (1.09 - 1.61) | (0.84 - 1.43) | (0.56 - 0.81) | (0.67 - 1.20) |
| Wave 3 | 0.99 | 1.22 | 1.04 | 0.87 |
|  | (0.82 - 1.19) | (0.95 - 1.56) | (0.87 - 1.23) | (0.66 - 1.15) |
| Wave 4 | 1.09 | 0.96 | 1.27*** | 1.03 |
|  | (0.94 - 1.27) | (0.79 - 1.16) | (1.10 - 1.46) | (0.82 - 1.28) |
| Wave 5 | 1.48*** | 1.04 | 0.71*** | 1.09 |
|  | (1.20 - 1.83) | (0.80 - 1.35) | (0.59 - 0.87) | (0.80 - 1.48) |
| Wave 6 | 1.21** | 0.98 | 0.85* | 1.29 |
|  | (1.00 - 1.47) | (0.76 - 1.25) | (0.71 - 1.02) | (0.98 - 1.70) |
| Cutpoint1 |  |  | 0.08*** | 0.01*** |
|  |  |  | (0.06 - 0.10) | (0.01 - 0.02) |
| Cutpoint2 |  |  | 0.16*** | 0.02*** |
|  |  |  | (0.12 - 0.22) | (0.02 - 0.04) |
| Cutpoint3 |  |  | 4.27*** | 9.85*** |
|  |  |  | (3.23 - 5.64) | (6.36 - 15.26) |
| Cutpoint4 |  |  | 25.61*** | 32.95*** |
|  |  |  | (18.98 - 34.57) | (20.80 - 52.20) |
| Constant | 5.16*** | 0.24*** |  |  |
|  | (3.81 - 6.99) | (0.17 - 0.36) |  |  |
| Observations | 5,982 | 5,970 | 5,900 | 5,719 |
| Pseudo R-squared | 0.0496 | 0.0572 | 0.00978 | 0.0189 |
| **Legend**: *** and ** Odds ratios are significant at the 1% and 5% significance level, respectively. 95% confidence intervals in parentheses. Ref: reference category | | | | |

Table S4: Full estimation: Logistic and ordinal logistic regressions of heavy episodic drinking, cannabis use at least once a week, and subjective change in alcohol and cannabis use on transgender and gender-diverse status with cisgender men as reference group.

|  | HED at once in the past week | Cannabis use at least once a week | Subjective change in alcohol use | Subjective change in cannabis use |
| --- | --- | --- | --- | --- |
| Transgender and gender-diverse | 1.78 | 3.20*** | 1.94* | 4.40*** |
|  | (0.75 - 4.26) | (1.60 - 6.41) | (0.98 - 3.85) | (2.04 - 9.49) |
| Cisgender women | 1.06 | 0.72*** | 0.95 | 0.93 |
|  | (0.95 - 1.19) | (0.62 - 0.84) | (0.85 - 1.06) | (0.79 - 1.11) |
| Cisgender men | Ref | Ref | Ref | Ref |
|  |  |  |  |  |
| Living with others | 0.99 | 1.22 | 1.04 | 0.87 |
|  | (0.82 - 1.19) | (0.95 - 1.56) | (0.87 - 1.23) | (0.66 - 1.15) |
| Presence of children | 1.09 | 0.96 | 1.27*** | 1.03 |
|  | (0.94 - 1.27) | (0.79 - 1.16) | (1.10 - 1.46) | (0.82 - 1.28) |
| High school | 0.48*** | 0.91 | 1.20** | 0.98 |
|  | (0.39 - 0.58) | (0.73 - 1.13) | (1.00 - 1.42) | (0.75 - 1.29) |
| Post-secondary | 1.05 | 1.16* | 1.00 | 0.98 |
|  | (0.92 - 1.18) | (0.98 - 1.38) | (0.89 - 1.13) | (0.81 - 1.18) |
| University | 1.25** | 1.05 | 0.94 | 0.79* |
|  | (1.05 - 1.47) | (0.83 - 1.33) | (0.81 - 1.10) | (0.61 - 1.03) |
| Asian | 0.95 | 0.51*** | 1.14* | 0.64*** |
|  | (0.82 - 1.09) | (0.42 - 0.61) | (1.00 - 1.30) | (0.52 - 0.79) |
| White | 0.64*** | 0.23*** | 0.81*** | 0.41*** |
|  | (0.54 - 0.75) | (0.18 - 0.30) | (0.70 - 0.95) | (0.32 - 0.52) |
| Urban | 0.98 | 1.42** | 1.19 | 1.41** |
|  | (0.79 - 1.22) | (1.05 - 1.91) | (0.97 - 1.45) | (1.02 - 1.95) |
| Rural | 0.65*** | 1.00 | 1.25*** | 1.44*** |
|  | (0.54 - 0.78) | (0.80 - 1.24) | (1.06 - 1.47) | (1.12 - 1.87) |
| Age 40-59 | 1.04 | 0.97 | 0.92 | 0.92 |
|  | (0.86 - 1.26) | (0.74 - 1.28) | (0.77 - 1.10) | (0.69 - 1.23) |
| Age 60+ | 1.01 | 1.02 | 0.90 | 0.93 |
|  | (0.84 - 1.22) | (0.78 - 1.33) | (0.75 - 1.07) | (0.69 - 1.24) |
| Separated | 1.27** | 0.94 | 0.72*** | 0.84 |
|  | (1.05 - 1.54) | (0.71 - 1.23) | (0.60 - 0.87) | (0.62 - 1.12) |
| Married | 1.19* | 1.34** | 0.80** | 1.32* |
|  | (0.99 - 1.45) | (1.04 - 1.74) | (0.66 - 0.96) | (1.00 - 1.76) |
| Wave 2 | 1.32*** | 1.10 | 0.67*** | 0.90 |
|  | (1.09 - 1.61) | (0.84 - 1.43) | (0.56 - 0.81) | (0.67 - 1.20) |
| Wave 3 | 0.99 | 1.22 | 1.04 | 0.87 |
|  | (0.82 - 1.19) | (0.95 - 1.56) | (0.87 - 1.23) | (0.66 - 1.15) |
| Wave 4 | 1.09 | 0.96 | 1.27*** | 1.03 |
|  | (0.94 - 1.27) | (0.79 - 1.16) | (1.10 - 1.46) | (0.82 - 1.28) |
| Wave 5 | 1.48*** | 1.04 | 0.71*** | 1.09 |
|  | (1.20 - 1.83) | (0.80 - 1.35) | (0.59 - 0.87) | (0.80 - 1.48) |
| Wave 6 | 1.21** | 0.98 | 0.85* | 1.29* |
|  | (1.00 - 1.47) | (0.76 - 1.25) | (0.71 - 1.02) | (0.98 - 1.70) |
| Cutpoint1 |  |  | 0.07*** | 0.01*** |
|  |  |  | (0.06 - 0.10) | (0.01 - 0.02) |
| Cutpoint2 |  |  | 0.15*** | 0.02*** |
|  |  |  | (0.12 - 0.21) | (0.01 - 0.04) |
| Cutpoint3 |  |  | 4.06*** | 9.21*** |
|  |  |  | (3.06 - 5.37) | (5.92 - 14.31) |
| Cutpoint4 |  |  | 24.34*** | 30.81*** |
|  |  |  | (17.98 - 32.93) | (19.40 - 48.94) |
| Constant | 4.86*** | 0.34*** |  |  |
|  | (3.58 - 6.59) | (0.23 - 0.49) |  |  |
| Observations | 5,982 | 5,970 | 5,900 | 5,719 |
| Pseudo R-squared | 0.0496 | 0.0572 | 0.00978 | 0.0189 |
| **Legend**: *** and ** Odds ratios are significant at the 1% and 5% significance level, respectively. 95% confidence intervals in parentheses. Ref: reference category | | | | |

Table S5: Sensitivity analysis: Logistic and ordinal logistic regressions of heavy episodic drinking, cannabis use at least once a week, and subjective change in alcohol and cannabis use on transgender or gender-diverse status with participants who did not answer the gender identity question.

| Gender groups | HED at least once in the past week | Cannabis use at least once a week | Subjective change in alcohol use | Subjective change in cannabis use |
| --- | --- | --- | --- | --- |
| Transgender and gender-diverse | 1.80 | 2.74*** | 2.16** | 3.35*** |
|  | (0.80 - 4.01) | (1.47 - 5.12) | (1.18 - 3.94) | (1.62 - 6.91) |
| Cisgender (women and men) | Ref | Ref | Ref | Ref |
| Transgender and gender-diverse | 1.74 | 3.23*** | 2.21** | 3.46*** |
|  | (0.78 - 3.90) | (1.72 - 6.07) | (1.21 - 4.04) | (1.67 - 7.17) |
| Cisgender men | 0.94 | 1.39*** | 1.05 | 1.07 |
|  | (0.84 - 1.05) | (1.19 - 1.62) | (0.95 - 1.17) | (0.90 - 1.27) |
| Cisgender women | Ref | Ref | Ref | Ref |
| Transgender and gender-diverse | 1.85 | 2.33*** | 2.10** | 3.23*** |
|  | (0.83 - 4.15) | (1.24 - 4.38) | (1.15 - 3.84) | (1.56 - 6.70) |
| Cisgender women | 1.06 | 0.72*** | 0.95 | 0.93 |
|  | (0.95 - 1.19) | (0.62 - 0.84) | (0.85 - 1.06) | (0.79 - 1.11) |
| Cisgender men | Ref | Ref | Ref | Ref |
| **Legend**: *** and ** Odds ratios are significant at the 1% and 5% significance level, respectively. 95% confidence intervals in parentheses. Ref: reference category. Subjective changes in alcohol use and changes in cannabis use variables are categorical with five categories: 1 (much less), 2 (slightly less), 3 (no change), 4 (slightly more), and 5 (much more). For these variables, proportional odds ratios were reported. Heavy episodic drinking and use of cannabis at least once a week are binary variables. **Note**: Odds ratios and 95% confidence intervals are adjusted for age, marital status, education, ethnicity, living area, the presence of children, other people in the household , and survey wave indicator variables. | | | | |

Table S6: Full estimation: Logistic and ordinal logistic regressions of heavy episodic drinking, cannabis use at least once a week, and subjective change in alcohol and cannabis use on transgender and gender-diverse status with cisgender participants (both men and women) as reference group. The six categories of age groups were used in this model.

|  | HED at least once in the past week | Cannabis use at least once a week | Subjective change in alcohol use | Subjective change in cannabis use |
| --- | --- | --- | --- | --- |
| Transgender and gender-diverse | 1.72 | 3.77*** | 1.98** | 4.56*** |
|  | (0.72 - 4.12) | (1.87 - 7.61) | (1.00 - 3.90) | (2.13 - 9.78) |
| Cisgender (men and women) | Ref. | Ref. | Ref. | Ref. |
| Living with others | 0.98 | 1.18 | 1.05 | 0.88 |
|  | (0.81 - 1.19) | (0.92 - 1.51) | (0.88 - 1.26) | (0.66 - 1.16) |
| Presence of children | 1.08 | 0.91 | 1.21** | 1.02 |
|  | (0.93 - 1.26) | (0.75 - 1.11) | (1.04 - 1.39) | (0.81 - 1.27) |
| High school | 1.48*** | 1.09 | 0.73*** | 1.10 |
|  | (1.20 - 1.84) | (0.84 - 1.41) | (0.60 - 0.89) | (0.81 - 1.50) |
| Post-secondary | 1.21* | 1.02 | 0.87 | 1.31* |
|  | (1.00 - 1.47) | (0.80 - 1.30) | (0.72 - 1.04) | (0.99 - 1.73) |
| University | 0.72*** | 0.62*** | 1.00 | 0.82* |
|  | (0.62 - 0.83) | (0.51 - 0.76) | (0.87 - 1.15) | (0.66 - 1.02) |
| Asian | 1.10 | 0.57*** | 0.88 | 0.98 |
|  | (0.87 - 1.39) | (0.43 - 0.75) | (0.72 - 1.08) | (0.72 - 1.35) |
| White | 0.48*** | 0.92 | 1.21** | 0.98 |
|  | (0.40 - 0.59) | (0.74 - 1.14) | (1.01 - 1.44) | (0.75 - 1.29) |
| Urban | 1.04 | 1.18* | 1.00 | 0.98 |
|  | (0.92 - 1.18) | (0.99 - 1.40) | (0.89 - 1.13) | (0.82 - 1.18) |
| Rural | 1.25*** | 1.02 | 0.93 | 0.79* |
|  | (1.06 - 1.48) | (0.80 - 1.29) | (0.80 - 1.09) | (0.61 - 1.01) |
| 30 to 39 years | 0.97 | 1.01 | 1.14 | 1.08 |
|  | (0.78 - 1.21) | (0.79 - 1.29) | (0.94 - 1.39) | (0.80 - 1.46) |
| 40 to 49 years | 0.95 | 0.63*** | 1.46*** | 0.69** |
|  | (0.75 - 1.21) | (0.47 - 0.85) | (1.17 - 1.82) | (0.48 - 0.97) |
| 50 to 59 years | 0.90 | 0.42*** | 1.09 | 0.67** |
|  | (0.71 - 1.13) | (0.31 - 0.57) | (0.88 - 1.36) | (0.48 - 0.94) |
| 60 to 69 years | 0.64*** | 0.30*** | 0.94 | 0.47*** |
|  | (0.51 - 0.81) | (0.22 - 0.41) | (0.75 - 1.18) | (0.33 - 0.66) |
| 70 years and over | 0.58*** | 0.13*** | 0.78** | 0.37*** |
|  | (0.45 - 0.75) | (0.08 - 0.21) | (0.61 - 1.00) | (0.25 - 0.56) |
| Separated | 1.01 | 1.45** | 1.20* | 1.41** |
|  | (0.81 - 1.26) | (1.07 - 1.96) | (0.97 - 1.47) | (1.01 - 1.95) |
| Married | 0.66*** | 1.05 | 1.24** | 1.44*** |
|  | (0.55 - 0.80) | (0.83 - 1.32) | (1.05 - 1.47) | (1.10 - 1.88) |
| Wave 2 | 1.04 | 0.97 | 0.92 | 0.93 |
|  | (0.86 - 1.26) | (0.74 - 1.27) | (0.77 - 1.11) | (0.69 - 1.24) |
| Wave 3 | 1.01 | 1.01 | 0.89 | 0.92 |
|  | (0.83 - 1.22) | (0.77 - 1.31) | (0.74 - 1.07) | (0.69 - 1.23) |
| Wave 4 | 1.27** | 0.93 | 0.72*** | 0.83 |
|  | (1.05 - 1.54) | (0.71 - 1.22) | (0.60 - 0.86) | (0.62 - 1.12) |
| Wave 5 | 1.19* | 1.32** | 0.79*** | 1.31* |
|  | (0.99 - 1.45) | (1.02 - 1.71) | (0.65 - 0.94) | (0.99 - 1.74) |
| Wave 6 | 1.32*** | 1.09 | 0.67*** | 0.89 |
|  | (1.09 - 1.61) | (0.83 - 1.42) | (0.56 - 0.80) | (0.67 - 1.20) |
| Cutpoint1 |  |  | 0.08*** | 0.01*** |
|  |  |  | (0.06 - 0.11) | (0.01 - 0.02) |
| Cutpoint2 |  |  | 0.17*** | 0.03*** |
|  |  |  | (0.13 - 0.24) | (0.02 - 0.04) |
| Cutpoint3 |  |  | 4.57*** | 10.12*** |
|  |  |  | (3.38 - 6.19) | (6.28 - 16.30) |
| Cutpoint4 |  |  | 27.52*** | 33.85*** |
|  |  |  | (19.92 - 38.01) | (20.59 - 55.66) |
| Constant | 5.10*** | 0.28*** |  |  |
|  | (3.65 - 7.13) | (0.19 - 0.42) |  |  |
| Observations | 5,982 | 5,970 | 5,900 | 5,719 |
| Pseudo R-squared | 0.0497 | 0.0585 | 0.0108 | 0.0192 |
| **Legend**: *** and ** Odds ratios are significant at the 1% and 5% significance level, respectively. 95% confidence intervals in parentheses. Ref: reference category | | | | |

Table S7: Full estimation: Logistic and ordinal logistic regressions of heavy episodic drinking, cannabis use at least once a week, and subjective change in alcohol and cannabis use on transgender and gender-diverse status with cisgender women as reference group. The six categories of age groups were used in this model.

|  | HED at least once in the past week | Cannabis use at least once a week | Subjective change in alcohol use | Subjective change in cannabis use |
| --- | --- | --- | --- | --- |
| Transgender and gender-diverse | 1.68 | 4.44*** | 2.02** | 4.71*** |
|  | (0.70 - 4.01) | (2.19 - 9.02) | (1.02 - 4.01) | (2.18 - 10.14) |
| Cisgender men | 0.94 | 1.40*** | 1.06 | 1.07 |
|  | (0.84 - 1.06) | (1.19 - 1.63) | (0.95 - 1.17) | (0.90 - 1.27) |
| Cisgender women | Ref. | Ref. | Ref. | Ref. |
| Living with others | 0.98 | 1.21 | 1.06 | 0.88 |
|  | (0.81 - 1.19) | (0.94 - 1.55) | (0.89 - 1.26) | (0.66 - 1.16) |
| Presence of children | 1.08 | 0.91 | 1.20** | 1.02 |
|  | (0.93 - 1.27) | (0.75 - 1.11) | (1.04 - 1.39) | (0.81 - 1.27) |
| High school | 1.49*** | 1.07 | 0.73*** | 1.10 |
|  | (1.20 - 1.84) | (0.82 - 1.39) | (0.60 - 0.89) | (0.80 - 1.49) |
| Post-secondary | 1.22** | 0.99 | 0.86 | 1.30* |
|  | (1.00 - 1.48) | (0.78 - 1.27) | (0.72 - 1.03) | (0.98 - 1.72) |
| University | 0.72*** | 0.61*** | 1.00 | 0.82* |
|  | (0.62 - 0.83) | (0.50 - 0.75) | (0.87 - 1.14) | (0.65 - 1.02) |
| Asian | 1.10 | 0.55*** | 0.88 | 0.98 |
|  | (0.87 - 1.40) | (0.42 - 0.73) | (0.72 - 1.08) | (0.71 - 1.34) |
| White | 0.48*** | 0.92 | 1.21** | 0.98 |
|  | (0.40 - 0.59) | (0.74 - 1.15) | (1.01 - 1.44) | (0.75 - 1.29) |
| Urban | 1.05 | 1.16 | 1.00 | 0.98 |
|  | (0.92 - 1.18) | (0.98 - 1.38) | (0.89 - 1.13) | (0.81 - 1.18) |
| Rural | 1.24** | 1.05 | 0.94 | 0.79 |
|  | (1.05 - 1.47) | (0.83 - 1.33) | (0.80 - 1.10) | (0.61 - 1.02) |
| 30 to 39 years | 0.98 | 0.97 | 1.14 | 1.07 |
|  | (0.78 - 1.21) | (0.76 - 1.24) | (0.93 - 1.38) | (0.79 - 1.45) |
| 40 to 49 years | 0.96 | 0.61*** | 1.45*** | 0.68** |
|  | (0.75 - 1.22) | (0.45 - 0.81) | (1.16 - 1.81) | (0.48 - 0.97) |
| 50 to 59 years | 0.90 | 0.40*** | 1.09 | 0.66** |
|  | (0.71 - 1.14) | (0.30 - 0.55) | (0.87 - 1.35) | (0.47 - 0.94) |
| 60 to 69 years | 0.65*** | 0.29*** | 0.94 | 0.46*** |
|  | (0.51 - 0.82) | (0.21 - 0.40) | (0.75 - 1.17) | (0.32 - 0.66) |
| 70 years and over | 0.58*** | 0.12*** | 0.77** | 0.37*** |
|  | (0.45 - 0.75) | (0.08 - 0.20) | (0.60 - 0.99) | (0.25 - 0.55) |
| Separated | 1.00 | 1.52*** | 1.21 | 1.42** |
|  | (0.80 - 1.24) | (1.12 - 2.06) | (0.98 - 1.49) | (1.02 - 1.97) |
| Married | 0.66*** | 1.04 | 1.24** | 1.43*** |
|  | (0.55 - 0.80) | (0.83 - 1.31) | (1.04 - 1.47) | (1.10 - 1.87) |
| Wave 2 | 1.04 | 0.97 | 0.92 | 0.93 |
|  | (0.86 - 1.26) | (0.74 - 1.28) | (0.77 - 1.11) | (0.69 - 1.24) |
| Wave 3 | 1.01 | 1.01 | 0.89 | 0.92 |
|  | (0.83 - 1.22) | (0.77 - 1.31) | (0.74 - 1.07) | (0.69 - 1.23) |
| Wave 4 | 1.27** | 0.93 | 0.72*** | 0.84 |
|  | (1.05 - 1.54) | (0.71 - 1.22) | (0.60 - 0.86) | (0.62 - 1.12) |
| Wave 5 | 1.19* | 1.33** | 0.79*** | 1.31 |
|  | (0.98 - 1.45) | (1.03 - 1.72) | (0.65 - 0.94) | (0.99 - 1.75) |
| Wave 6 | 1.32*** | 1.09 | 0.67*** | 0.89 |
|  | (1.09 - 1.61) | (0.83 - 1.42) | (0.56 - 0.80) | (0.67 - 1.20) |
| Cutpoint1 |  |  | 0.09*** | 0.01*** |
|  |  |  | (0.06 - 0.12) | (0.01 - 0.02) |
| Cutpoint2 |  |  | 0.18*** | 0.03*** |
|  |  |  | (0.13 - 0.24) | (0.02 - 0.04) |
| Cutpoint3 |  |  | 4.66*** | 10.38*** |
|  |  |  | (3.43 - 6.33) | (6.42 - 16.80) |
| Cutpoint4 |  |  | 28.07*** | 34.75*** |
|  |  |  | (20.27 - 38.87) | (21.04 - 57.37) |
| Constant | 5.22*** | 0.24*** |  |  |
|  | (3.72 - 7.31) | (0.16 - 0.37) |  |  |
| Observations | 5,982 | 5,970 | 5,900 | 5,719 |
| Pseudo R-squared | 0.0498 | 0.0623 | 0.0109 | 0.0193 |
| **Legend**: *** and ** Odds ratios are significant at the 1% and 5% significance level, respectively. 95% confidence intervals in parentheses. Ref: reference category | | | | |

Table S8: Full estimation: Logistic and ordinal logistic regressions of heavy episodic drinking, cannabis use at least once a week, and subjective change in alcohol and cannabis use on transgender and gender-diverse status with cisgender men as reference group. The six categories of age groups were used in this model.

|  | HED at least once in the past week | Cannabis use at least once a week | Subjective change in alcohol use | Subjective change in cannabis use |
| --- | --- | --- | --- | --- |
| Transgender and gender-diverse | 1.78 | 3.18*** | 1.92 | 4.40*** |
|  | (0.74 - 4.25) | (1.57 - 6.46) | (0.97 - 3.80) | (2.04 - 9.48) |
| Cisgender women | 1.06 | 0.72*** | 0.95 | 0.93 |
|  | (0.95 - 1.19) | (0.61 - 0.84) | (0.85 - 1.05) | (0.79 - 1.11) |
| Cisgender men | Ref. | Ref. | Ref. | Ref. |
| Living with others | 0.98 | 1.21 | 1.06 | 0.88 |
|  | (0.81 - 1.19) | (0.94 - 1.55) | (0.89 - 1.26) | (0.66 - 1.16) |
| Presence of children | 1.08 | 0.91 | 1.20** | 1.02 |
|  | (0.93 - 1.27) | (0.75 - 1.11) | (1.04 - 1.39) | (0.81 - 1.27) |
| High school | 1.49*** | 1.07 | 0.73*** | 1.10 |
|  | (1.20 - 1.84) | (0.82 - 1.39) | (0.60 - 0.89) | (0.80 - 1.49) |
| Post-secondary | 1.22** | 0.99 | 0.86 | 1.30* |
|  | (1.00 - 1.48) | (0.78 - 1.27) | (0.72 - 1.03) | (0.98 - 1.72) |
| University | 0.72*** | 0.61*** | 1.00 | 0.82* |
|  | (0.62 - 0.83) | (0.50 - 0.75) | (0.87 - 1.14) | (0.65 - 1.02) |
| Asian | 1.10 | 0.55*** | 0.88 | 0.98 |
|  | (0.87 - 1.40) | (0.42 - 0.73) | (0.72 - 1.08) | (0.71 - 1.34) |
| White | 0.48*** | 0.92 | 1.21** | 0.98 |
|  | (0.40 - 0.59) | (0.74 - 1.15) | (1.01 - 1.44) | (0.75 - 1.29) |
| Urban | 1.05 | 1.16* | 1.00 | 0.98 |
|  | (0.92 - 1.18) | (0.98 - 1.38) | (0.89 - 1.13) | (0.81 - 1.18) |
| Rural | 1.24** | 1.05 | 0.94 | 0.79 |
|  | (1.05 - 1.47) | (0.83 - 1.33) | (0.80 - 1.10) | (0.61 - 1.02) |
| 30 to 39 years | 0.98 | 0.97 | 1.14 | 1.07 |
|  | (0.78 - 1.21) | (0.76 - 1.24) | (0.93 - 1.38) | (0.79 - 1.45) |
| 40 to 49 years | 0.96 | 0.61*** | 1.45*** | 0.68** |
|  | (0.75 - 1.22) | (0.45 - 0.81) | (1.16 - 1.81) | (0.48 - 0.97) |
| 50 to 59 years | 0.90 | 0.40*** | 1.09 | 0.66** |
|  | (0.71 - 1.14) | (0.30 - 0.55) | (0.87 - 1.35) | (0.47 - 0.94) |
| 60 to 69 years | 0.65*** | 0.29*** | 0.94 | 0.46*** |
|  | (0.51 - 0.82) | (0.21 - 0.40) | (0.75 - 1.17) | (0.32 - 0.66) |
| 70 years and over | 0.58*** | 0.12*** | 0.77** | 0.37*** |
|  | (0.45 - 0.75) | (0.08 - 0.20) | (0.60 - 0.99) | (0.25 - 0.55) |
| Separated | 1.00 | 1.52*** | 1.21* | 1.42** |
|  | (0.80 - 1.24) | (1.12 - 2.06) | (0.98 - 1.49) | (1.02 - 1.97) |
| Married | 0.66*** | 1.04 | 1.24** | 1.43*** |
|  | (0.55 - 0.80) | (0.83 - 1.31) | (1.04 - 1.47) | (1.10 - 1.87) |
| Wave 2 | 1.04 | 0.97 | 0.92 | 0.93 |
|  | (0.86 - 1.26) | (0.74 - 1.28) | (0.77 - 1.11) | (0.69 - 1.24) |
| Wave 3 | 1.01 | 1.01 | 0.89 | 0.92 |
|  | (0.83 - 1.22) | (0.77 - 1.31) | (0.74 - 1.07) | (0.69 - 1.23) |
| Wave 4 | 1.27** | 0.93 | 0.72*** | 0.84 |
|  | (1.05 - 1.54) | (0.71 - 1.22) | (0.60 - 0.86) | (0.62 - 1.12) |
| Wave 5 | 1.19* | 1.33** | 0.79*** | 1.31 |
|  | (0.98 - 1.45) | (1.03 - 1.72) | (0.65 - 0.94) | (0.99 - 1.75) |
| Wave 6 | 1.32*** | 1.09 | 0.67*** | 0.89 |
|  | (1.09 - 1.61) | (0.83 - 1.42) | (0.56 - 0.80) | (0.67 - 1.20) |
| Cutpoint1 |  |  | 0.08*** | 0.01*** |
|  |  |  | (0.06 - 0.11) | (0.01 - 0.02) |
| Cutpoint2 |  |  | 0.17*** | 0.02*** |
|  |  |  | (0.12 - 0.23) | (0.01 - 0.04) |
| Cutpoint3 |  |  | 4.42*** | 9.70*** |
|  |  |  | (3.24 - 6.03) | (5.95 - 15.80) |
| Cutpoint4 |  |  | 26.60*** | 32.45*** |
|  |  |  | (19.12 - 37.00) | (19.53 - 53.93) |
| Constant | 4.93*** | 0.34*** |  |  |
|  | (3.50 - 6.93) | (0.23 - 0.52) |  |  |
| Observations | 5,982 | 5,970 | 5,900 | 5,719 |
| Pseudo R-squared | 0.0498 | 0.0623 | 0.0109 | 0.0193 |
| **Legend**: *** and ** Odds ratios are significant at the 1% and 5% significance level, respectively. 95% confidence intervals in parentheses. Ref: reference category | | | | |
