# Supplementary material for: Alcohol and cannabis use during the COVID-19 pandemic among transgender, gender-diverse, and cisgender adults in Canada
Source: BMC Public Health. 2022 Mar 7;22:452. doi: 10.1186/s12889-022-12779-9 (PMC8899793; doi:10.1186/s12889-022-12779-9)
Supplement: Supplementary file 2 — Additional file 2. Survey Questionnaire. [file 12889_2022_12779_MOESM2_ESM.pdf]

Survey Questionnaire

Examining the Impact of COVID-19 on Mental Health and
Substance Use among Canadians

Date of Survey: (month, day, year) auto time stamp

Time of Survey: auto time stamp

1. In which province or territory do you currently live?
   - o Alberta
   - o British Columbia
   - o Manitoba
   - o New Brunswick
   - o Newfoundland and Labrador
   - o Northwest Territories
   - o Nova Scotia
   - o Nunavut
   - o Ontario
   - o Prince Edward Island
   - o Quebec
   - o Saskatchewan
   - o Yukon

   - o I currently live outside of Canada

{*NOTE: Some web panel members might have moved outside of Canada. Respondents who indicate that they currently live outside of Canada will be excluded/exited from the survey*.}

2. To which of the following age groups do you belong?

   - o 18 to 29 years
   - o 30 to 39 years
   - o 40 to 49 years
   - o 50 to 59 years
   - o 60 to 69 years
   - o 70 years and over

   - o Prefer not to answer

3. How do you describe your gender identity?

- Man
- Woman
- Transgender man
- Transgender woman
- Two-Spirit
- Non-binary (genderqueer, gender fluid)
- Questioning/Not sure of my gender identity
- Identity not listed
- Prefer not to answer

The next few questions are related to the COVID-19 pandemic.

4. Have you, or those close to you (e.g., close relative/friend), tested positive for COVID-19 or are at high risk of COVID-19? (check all that apply)

- I, or someone close to me, has tested positive for COVID-19
- I, or someone close to me, has had symptoms of COVID-19 but has not been tested
- I, or someone close to me, has been tested for COVID-19 but it was negative (i.e., they did not have COVID-19)
- I, or someone close to me, is elderly and/or has a health condition that increases the risk of serious illness from COVID-19
- I have a job that exposes me to high risk of getting COVID-19
- Someone close to me has a job that exposes them to high risk of getting COVID-19
- None of the above

5. How worried are you about the impact of COVID-19 on your personal financial situation?

- Very worried
- Somewhat worried
- Not very worried
- Not at all worried

6. How have physical distancing measures due to the COVID-19 pandemic affected your employment situation? (check one only)

- I have continued working, but now I am working from home instead of my usual location
- I am not currently working, or I have been laid off/let go, due to the pandemic (***Skip to Question 8***)
- I was working from home due to the pandemic, but now I am back working at my usual location outside the home

- I was previously not working/ laid off/let go due to the pandemic, but now I am back at work with the same or a new employer
- No change - I have continued working outside my home, as I always did
- No change - I have continued working from home, as I always did
- No change - I was not employed prior to the pandemic (e.g., retired, student, paid leave, recently graduated) and I have remained unemployed (***Skip to Question 8***)
- Other

7. On average, how has the number of hours you are working for pay been affected by the COVID-19 pandemic?

- Increased a lot
- Increased somewhat
- No change
- Decreased somewhat
- Decreased a lot

8. How worried are you that you or someone close to you (close relative or friend) will get ill from COVID-19?

- Very worried
- Somewhat worried
- Not very worried
- Not at all worried

The next few questions are about how you have been feeling lately.

Over the PAST 2 WEEKS, how often have you been bothered by the following problems?

9. Feeling nervous, anxious or on edge
- Not at all
- Several days
- Over half the days
- Nearly every day

10. Not being able to stop or control worrying
- Not at all
- Several days
- Over half the days
- Nearly every day

11. Worrying too much about different things
- Not at all
- Several days

- Over half the days
- Nearly every day

12. Trouble relaxing
    - Not at all
    - Several days
    - Over half the days
    - Nearly every day

13. Being so restless that it's hard to sit still
    - Not at all
    - Several days
    - Over half the days
    - Nearly every day

14. Becoming easily annoyed or irritable
    - Not at all
    - Several days
    - Over half the days
    - Nearly every day

15. Feeling afraid as if something awful might happen

    - Not at all
    - Several days
    - Over half the days
    - Nearly every day

The next few questions are about alcohol and cannabis.

16. During the <u>PAST 7 DAYS</u>, on how many days did you drink ALCOHOL?

    __ Number of days
    - I do not drink alcohol

    - Prefer not to answer

{Note: If response is 0 days or "I do not drink alcohol", SKIP Q17}

17. On how many of the PAST 7 DAYS did you drink [4 (if woman) or 5 (if man) or 5 (if other gender)] or more drinks on one occasion? A drink means a 341 ml or 12 oz. bottle of beer or cider/cooler (5% alcohol content), a 142 ml or 5 oz. glass of wine (12% alcohol content), or a straight or mixed drink with 43 ml or 1.5 oz. of liquor (40% alcohol content – e.g., rye, gin, rum).

__ Number of days

- Prefer not to answer

18. In the PAST 7 DAYS, did you drink more ALCOHOL, about the same, or less alcohol overall than you did before the COVID-19 pandemic started?

- Drink much more alcohol
- Drink slightly more alcohol
- No change
- Drink slightly less alcohol
- Drink much less alcohol

- Prefer not to answer

19. During the PAST 7 DAYS, on how many days did you use CANNABIS (also known as marijuana, hash, "pot")?

__ Number of days
- I do not use cannabis

- Prefer not to answer

20. In the PAST 7 DAYS, did you use CANNABIS more often, about the same, or less often overall than you did before the COVID-19 pandemic started?

- Much more
- Slightly more
- No change
- Much less
- Slightly less

- Prefer not to answer

Now, we would like to ask you some questions about how you have been feeling over the past 7 days.

21. In the PAST 7 DAYS, how often have you felt depressed?

- o Rarely or none of the time (less than 1 day)
- o Some or a little of the time (1-2 days)
- o Occasionally or a moderate amount of the time (3-4 days)
- o Most or all of the time (5-7 days)

22. In the PAST 7 DAYS, how often have you felt lonely?

- o Rarely or none of the time (less than 1 day)
- o Some or a little of the time (1-2 days)
- o Occasionally or a moderate amount of the time (3-4 days)
- o Most or all of the time (5-7 days)

23. In the PAST 7 DAYS, how often have you felt hopeful about the future?

- o Rarely or none of the time (less than 1 day)
- o Some or a little of the time (1-2 days)
- o Occasionally or a moderate amount of the time (3-4 days)
- o Most or all of the time (5-7 days)

The next few questions are about yourself and your household.

24. Including yourself, how many people are currently living in your household?

___ Enter number

- o Prefer not to answer

25. How many children in each of the following categories live in your household?

- o Under 6 years old : ___ Enter number
- o 6-12 years old: ___ Enter number
- o 13-17 years old: ___ Enter number

- o Prefer not to answer

26. What is the highest level of education you have completed?

- Did not graduate from high school
- Completed high school
- Some post-high school education (college, technical, university, etc.)
- College diploma / degree
  University diploma / degree

- Prefer not to answer

27. What is your current marital status?

- Married
- Living with a partner
- Widowed
- Divorced
- Separated
- Never married

- Prefer not to answer

28. Which of the following best describes your racial or ethnic group? (Check one only)

- Asian – East (e.g., Chinese, Japanese, Korean)
- Asian – South (e.g., Indian, Pakistani, Sri Lankan)
- Asian – South East (e.g., Malaysian, Filipino, Vietnamese)
- Black (Africa, Caribbean, North American)
- Indigenous (First Nations, Inuit, Métis)
- Latin American (e.g., Argentinean, Chilean, Salvadoran)
- Middle Eastern (e.g., Egyptian, Iranian, Lebanese)
- White (European, North American)
- Mixed heritage (e.g. Black – North American & White – North American)
- Other

- Not sure
- Prefer not to answer

29. What is the total household income you and other members of your household received in the year ending December 31st, 2019 before taxes? Please include income FROM ALL SOURCES such as savings, pensions, rent, and unemployment insurance as well as wages.

- less than $20,000

- $20,000 - $39,999
- $40,000 - $59,999
- $60,000 - $79,999
- $80,000 - $99,999
- $100,000 - $119,999
- $120,000 - $139,999
- $140,000 or more

- Prefer not to answer

30. Do you consider yourself to be living in a…

- Urban area
- Suburban area
- Rural area

Thank you for your time.
